# Citicoline Modifies the Expression of Specific miRNAs Related to Cardioprotection in Patients with ST-Segment Elevation Myocardial Infarction Subjected to Coronary Angioplasty

**DOI:** 10.3390/ph15080925

**Published:** 2022-07-27

**Authors:** Alejandro Silva-Palacios, Miguel Arroyo-Campuzano, Mirthala Flores-García, Mariana Patlán, Adrián Hernández-Díazcouder, Diego Alcántara, Ixchel Ramírez-Camacho, Dana Arana-Hidalgo, Elizabeth Soria-Castro, Fausto Sánchez, Héctor González-Pacheco, Cecilia Zazueta

**Affiliations:** 1 Departamento de Biomedicina Cardiovascular, Instituto Nacional de Cardiología Ignacio Chávez, Juan Badiano No. 1, Colonia Sección XVI, México City 14080, Mexico; alejandro.silva@cardiologia.org.mx (A.S.-P.); arroyocampuzanomiguel@gmail.com (M.A.-C.); diego123alcantara@hotmail.com (D.A.); ixch2010@gmail.com (I.R.-C.); fanyah20@gmail.com (D.A.-H.); elizabeth.soria@cardiologia.org.mx (E.S.-C.); 2 Departamento de Biología Molecular, Instituto Nacional de Cardiología, Ignacio Chávez, Juan Badiano No. 1, Colonia Sección XVI, México City 14080, Mexico; mirthala.flores@cardiologia.org.mx; 3 Subdirección de Investigación Básica y Tecnológica, Instituto Nacional de Cardiología, Ignacio Chávez, Juan Badiano No. 1, Colonia Sección XVI, México City 14080, Mexico; mariana.patlan@cardiologia.org.mx; 4 Departamento de Inmunología, Instituto Nacional de Cardiología, Ignacio Chávez, Juan Badiano No. 1, Colonia Sección XVI, México City 14080, Mexico; adrian.hernandez.diazc@hotmail.com (A.H.-D.); fausto.sanchez@cardiologia.org.mx (F.S.); 5 Unidad Coronaria, Instituto Nacional de Cardiología Ignacio Chávez, México City 14080, Mexico; hectorglzp@hotmail.com

**Keywords:** exosomes, STEMI, citicoline, caveolin-1, caveolin-3, hnRNPA2B1, miRNAs, reperfusion therapy, cardiac biomarker

## Abstract

Extracellular vesicles are recognized as signaling mediators between cells both in physiological and pathological communication. In this work, we explored the potential effect of citicoline to modify relevant proteins or miRNAs for cardioprotection in the smallest population of such microvesicles; i.e., in exosomes from patients diagnosed with ST-segment elevation myocardial infarction (STEMI) undergoing coronary angioplasty. The plasma-exosome-enriched fraction from these patients was characterized. Their cellular origin was assessed by flow cytometry and Western blot, whereas miRNA expression was evaluated by real-time polymerase chain reaction (qRT-PCR). The content of caveolin-1, caveolin-3, and hnRNPA2B1, which play a relevant role in selective transport of miRNAs into microvesicles, along with the effect on cell viability of the exosomes obtained from citicoline-treated and untreated groups were also analyzed. Our results showed that hypoxic stress increases exosome release into the circulation. Moreover, we found that CD146+ increased in exosomes from citicoline-treated patients, while CD142+ decreased in these patients compared to the placebo group. No changes were detected in the protein levels of caveolin-1, caveolin-3, and hnRNPA2B1. Citicoline administration modified the expression of miR233-3p, miR92, and miR21-5p in exosomes. Cell viability decreased in the presence of exosomes from infarcted patients, while incubation of H9c2 cells with exosomes from patients reperfused with citicoline did not affect cell viability. In conclusion, citicoline administration modifies the expression of specific miRNAs related to cardioprotection in exosomes.

## 1. Introduction

Extracellular vesicles (EVs) are small membranous bodies containing lipids, proteins, and non-coding RNAs of various origins and sizes. Larger vesicles are apoptotic bodies containing apoptotic nuclear material with diameters higher than 1 μm in diameter [1]. Microvesicles (MVs, also known as microparticles or ectosomes) have diameters between 100 and 1000 nm, which arise from the plasma membrane and express surface-specific antigens from their parent cell. Exosomes are the smallest particles (around 40–100 nm) that are released after the fusion of an intermediate endocytic compartment with the plasma membrane of many cell types [2]. These vesicles express characteristic markers, such as CD9, CD63, and CD81, as well as markers of the cells from which they originated, forming typical molecular signatures [3]. Exosomes play a key role in extracellular signaling communication, delivering lipids, and genetic material [4,5], besides cytosolic and membrane proteins to other cells [6]. The study of their mechanisms of action and their possible role in diagnostic and therapeutic applications in several pathologies, particularly in coronary artery disease, constitutes a novel and dynamic field of research [7], because the content of such particles may change in response to stress or injury.

Pathological-induced stress during myocardial reperfusion damage profoundly affects both endothelial cells and cardiomyocytes, so it is speculated that delivery of exosomes by endothelial cells (or other cell types) might constitute a “crosstalk” link between different cells in such conditions [8]. In this regard, it is known that endothelial microparticles increase in patients with acute coronary syndrome (ACS) [9], that exosomes containing different myocardial microRNAs (miRNAs) are released into the circulation after acute infarction [10], and that different proteins are contained in EVs from patients with ACS compared to subjects without ACS [11].

EVs may be prognostic markers in patients at risk to develop adverse cardiovascular outcomes and, also, are signaling mediators between cells during stress. To our knowledge, there is scarce evidence of changes in exosome-derived proteins or miRNAs associated with cardioprotective strategies in patients. In this sense, the possible protective effect of citicoline in patients was hypothesized from results obtained previously in pre-clinical models of reperfusion damage, in which this compound was shown to diminish oxidative stress, apoptosis, inflammation, and to inhibit the mitochondrial permeability transition pore, all of them being triggers of reperfusion damage [12,13,14,15]. Citicoline is the international nonproprietary name of cytidine-5′-diphosphocholine, which according to double-blind, placebo-controlled trials is moderately effective in alleviating cognitive and behavioral conditions in patients with chronic cerebral disorders, e.g., stroke [16], Parkinson’s disease [17], and Alzheimer’s disease [18]. Administration of citicoline increases brain choline, augments phosphatidylcholine synthesis, decreases free fatty acids levels, and reduces oxidative stress [19]. Therefore, in this work we explored whether citicoline modifies exosome content in patients with acute myocardial infarction (AMI) undergoing coronary angioplasty, to evaluate if these vesicles might be transmitters of cardioprotective molecules.

## 2. Results

Characteristics of patients admitted to the intensive care unit of the Instituto Nacional de Cardiología (México City, Mexico) suffering from STEMI and candidates for primary angioplasty are shown in Table 1. The patients, who were around 55 years old, randomly received placebo or citicoline at the time of reperfusion. Most of the participants were men with overweight or obesity and the main affected vessels were the left ascending and right coronary arteries.

Table 1 Characteristics and risk factors of the fifty-six patients admitted to the intensive care unit of the Instituto Nacional de Cardiología (Mexico City, Mexico) suffering from ST-segment elevation myocardial infarct (STEMI) and candidates for primary angioplasty. Half of such patients randomly received 2 g per day of citicoline: 1 g at the time of reperfusion and then 1000 mg every 12 h by intravenous infusion for the first 5 days, whereas the other patients received placebo.

**Figure 1:**
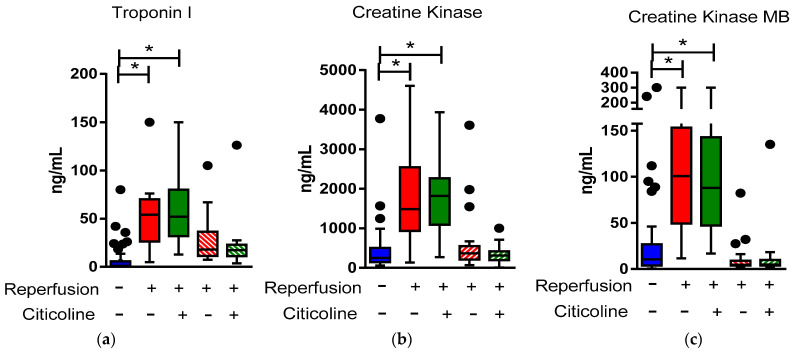
Box and whisker plot (Tukey Method, outliers in black dots) illustrates the total distribution of enzymatic necrosis markers before (AMI) and after primary angioplasty with and without citicoline: (**a**) Troponin-I, (**b**) creatine kinase, and (**c**) creatine kinase MB values before and after primary angioplasty at 24 h (solid colors) and at 72 h (dashed colors) in patients with ST-myocardial infarction (STEMI), *n* = 56, who received placebo (*n* = 28) or citicoline (*n* = 28). *p* value was determined using one-way ANOVA. * *p* < 0.001.

Myocardial necrosis biomarkers, troponin I, creatine kinase, and creatine kinase MB content increased in ranges of (7.8–150 ng/mL), (421.4–4601 ng/mL), and (11.4–300 ng/mL), respectively, 24 h after reperfusion (Figure 1a–c). Such values diminished after 72 h of reperfusion in all cases; although, troponin I content remain slightly higher than in those samples taken at the income time (Figure 1a). We did not evaluate final clinical outcomes in these patients due to the small number and the relatively short time of follow-up of many of them. Analysis of multiple risk factors and survival will be performed when a larger number of patients are recruited; these results would be part of another report. However, during hospitalization, we noticed that adverse events such as acute heart failure, ventricular arrhythmias, and all-cause mortality were more frequent in the placebo group (6.9% vs. 3.7%, 10.3% vs. 3.7%, and 6.9% vs. 0%, respectively) without significant differences.

### 2.1. Characterization of Exosome-Enriched Fractions

**Figure 2:**
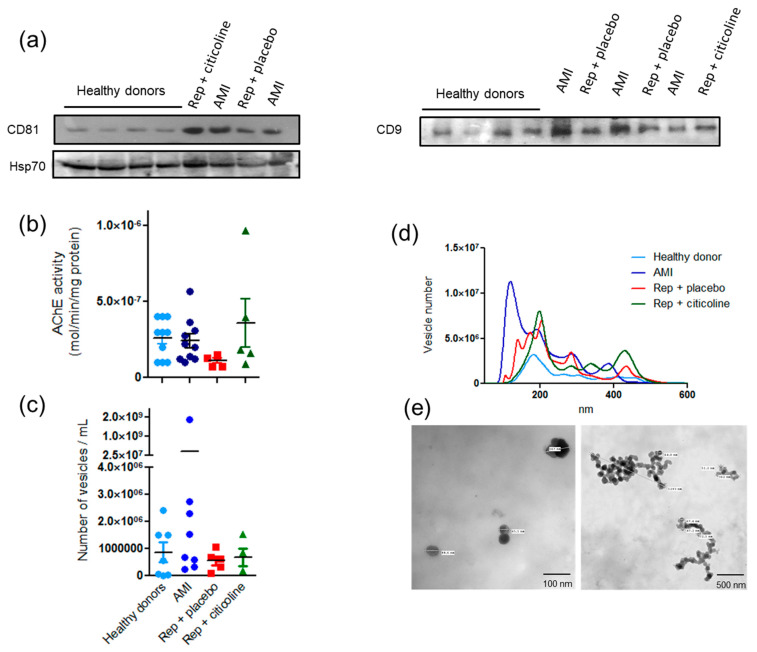
Characterization of exosomes from plasma of healthy donors and plasma of AMI patients before and after coronary angioplasty in presence of citicoline: (**a**) Western-blot analysis of tetraspanins CD9, CD81, and Hsp70. (**b**) Acetylcholine esterase activity. Results shown are the mean ± SE of at least 8 independent exosome samples from each group. (**c**) Quantification of particles number with size between 50 and 100 nm by nanoparticle tracking analysis (NTA), results are the mean ± SE of at least 5 independent exosomes’ samples from each group). (**d**) Representative image of particles size distribution between 50 and 600 nm determined by NTA. (**e**) Representative transmission electron microscopy images of exosome samples of the different groups.

Exosomes obtained with the ExoQuick^®^ kit from plasma samples were analyzed by different criteria. Typical markers CD9 and CD81 were evaluated by Western blot in exosomes from both control and infarcted patients. Interestingly, the amount of CD81 increased in the infarcted group and remained elevated before and after reperfusion. We also measured Hsp70 content, as a specific marker of cardiomyocyte-derived exosomes (Figure 2a). No differences were found when measuring acetylcholinesterase (AChE) activity, an enzymatic marker of exosomes. AChE activity was 2.6 × 10^−7^ mol of hydrolyzed substrate/min/mg protein in healthy donors, 2.2 × 10^−7^ mol of hydrolyzed substrate/min/mg in AMI patients, 1.13 × 10^−7^ mol of hydrolyzed substrate/min/mg in reperfused patients that received placebo, and 3.6 × 10^−7^ mol of hydrolyzed substrate/min/mg in reperfused patients treated with citicoline (Figure 2b). On the other hand, NTA analysis revealed different patterns of exosome samples in both total vesicle content (Figure 2c) and particle size (Figure 2d) among groups.

Increased number of vesicles of size range between 50 and 500 nm was observed in infarcted patients before reperfusion, as compared with particles obtained from control subjects and in samples obtained after coronary angioplasty with or without citicoline (Figure 2d). TEM analysis revealed that purified exosomes’ preparations include both individual vesicles and aggregates of the expected size and morphology (Figure 1e), confirming the enrichment of exosomes and showing that high-size particles represent an aggregated of such particles. Overall, our results indicate that conditions of hypoxic stress increase exosomes’ release into the circulation. Although the increase in blood exosomes might be a reliable marker to reinforce early diagnosis of cardiac injury in STEMI patients, such an increase is not maintained after reperfusion, which leads us to think that exosome release is not related to further necrotic death.

### 2.2. Exosomes Secretion from Different Cellular Types

**Figure 3:**
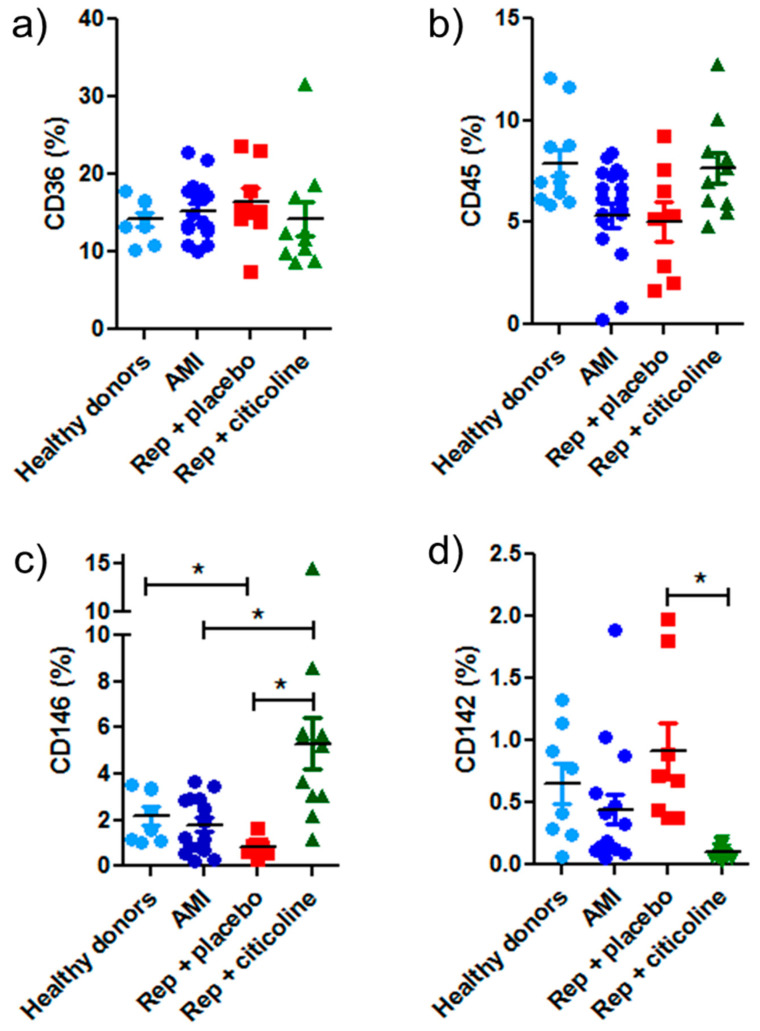
Flow cytometric analysis of exosomes obtained from plasma of AMI patients subjected to coronary angioplasty and treated with citicoline. Histograms showing mean fluorescence intensity of (**a**) CD36+, (**b**) CD45+, (**c**) CD146+, and (**d**) CD142+ antibodies in exosomes from healthy donors, from AMI patients, and from those subjected to angioplasty without (Rep + placebo) or with citicoline treatment. Data are expressed as mean ± SD of at least 10 exosome samples from different patients of each group. * *p* ≤ 0.05.

Besides determining the exosome features at the single-particle level, we characterized them according to their possible cellular origin. Exosomes from healthy donors and patients expressed mainly the fatty acid transporter CD36 over other evaluated cellular markers, e.g., 15–18% vs. CD45 (8%), CD146 (5%), and CD142 (1%) (Figure 3a). CD45, a receptor-like tyrosine phosphatase expressed at high levels on the surface of all nucleated hematopoietic cells, was expressed similarly in exosome samples from healthy donors and patients (Figure 3b). On the other hand, CD146 (a cell adhesion molecule expressed in all types of human endothelial cells) significantly increased in exosomes from patients treated with citicoline during the reperfusion procedure in comparison with exosomes from reperfused patients that received placebo and with exosomes obtained before angioplasty. Low levels of this molecule were also observed in exosomes from healthy donors (Figure 3c). Conversely, exosomes carrying tissue factor (CD142) diminished significantly in reperfused patients treated with citicoline, as compared with the group that received a placebo (Figure 3d). As described above, Hsp70 was detected in all samples, denoting the presence of a subpopulation of cardiomyocyte-derived exosomes (Figure 2a).

### 2.3. Citicoline Modifies miR-233-3p, miR21-5p, and miR-92 levels in Exosomes from Infarcted Patients Subjected to Angioplasty

**Figure 4:**
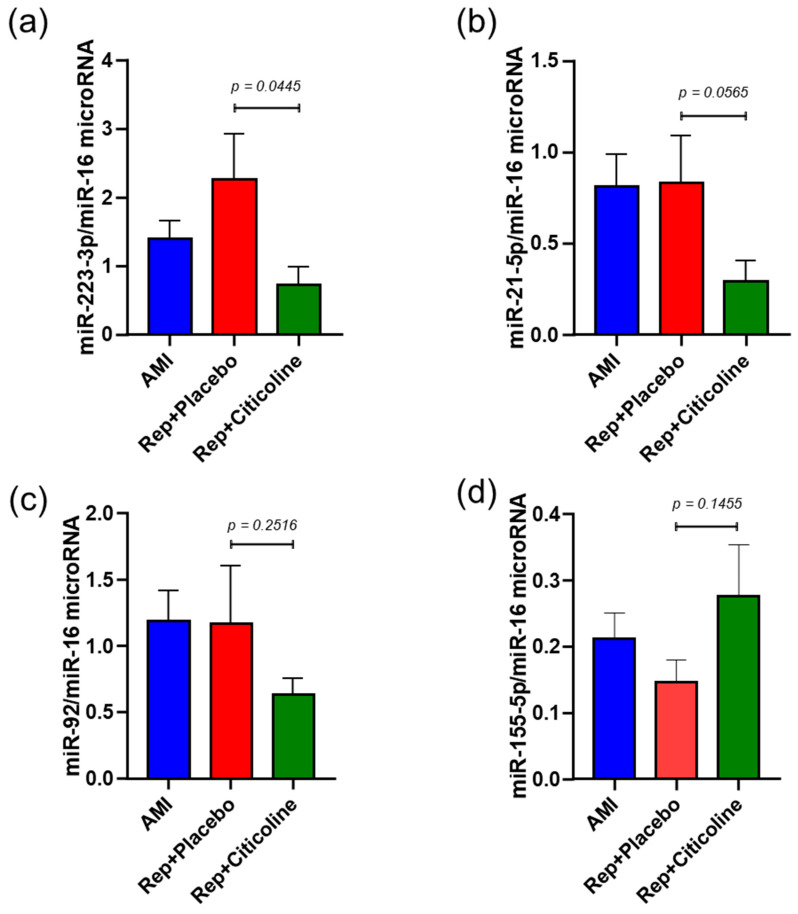
Differentially expressed miRNAs transcripts in exosomes obtained from plasma of AMI patients subjected to coronary angioplasty and treated with citicoline. Relative concentrations of (**a**) miR-223-3p, (**b**) miR-21-5p, (**c**) miR-92, (**d**) miR-155-5p were normalized with values of cel-miR-16 content and values were calculated using the 2−ΔCt formula. Results are shown as mean ± SD of at least 10 exosome samples from different patients of each group and were analyzed with the Mann–Whitney test.

miR-223-3p, miR21-5p, miR-92, and miR155-5p were measured by a specific stem-loop real-time polymerase chain reaction and normalized with miR-16. We found that both miR-233-3p, miR21-5p, and miR-92 (Figure 4a,c) were significantly reduced in exosomes obtained from reperfused patients treated with citicoline in comparison with the samples evaluated from patients that received placebo (*p* = 0.0445, *p* = 0.0565 and *p* = 0.2516, respectively). In the case of exosomes without treatment, all miRNAs were similar to those observed in exosomes from patients before the angioplasty procedure (Figure 4a–d). Conversely, compared with reperfused patients without treatment, levels of miR-155-5p increased significantly in exosomes from the group in which citicoline was administrated (*p* = 0.1455).

### 2.4. Caveolin and hnRNPA2B1 Content in Exosomes from Infarcted Patients Subjected to Angioplasty

**Figure 5:**
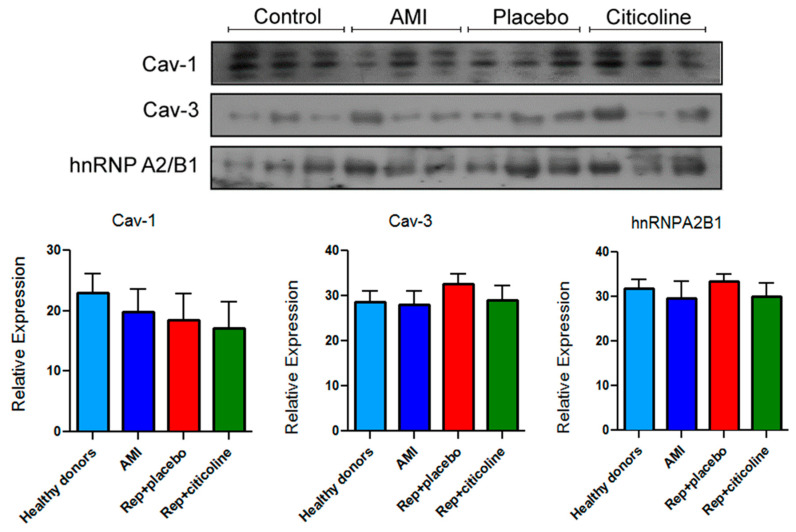
Immunodetection of proteins related to RNA trafficking process into exosomes. Representative images of Caveolin-1 (Cav-1), Caveolin-3 (Cav-3), and hnRNPA2B1 content as well as their densitometric analysis. Results are shown as mean ±SEM of exosome samples from different patients in each group (*n* = 9).

To evaluate possible changes in proteins that play a role in the selective transport of miRNAs into these and other microvesicles, we measured Cav-3, Cav-1, and hnRNPA2B1 content in exosome samples. As shown in the representative image and densitometric analysis, we did not detect differences in any of the proteins evaluated in the samples obtained from different patients of the three groups (Figure 5).

### 2.5. Effect of the Incubation of Exosomes from Reperfused Patients Treated with Citicoline in H9c2 Viability

**Figure 6:**
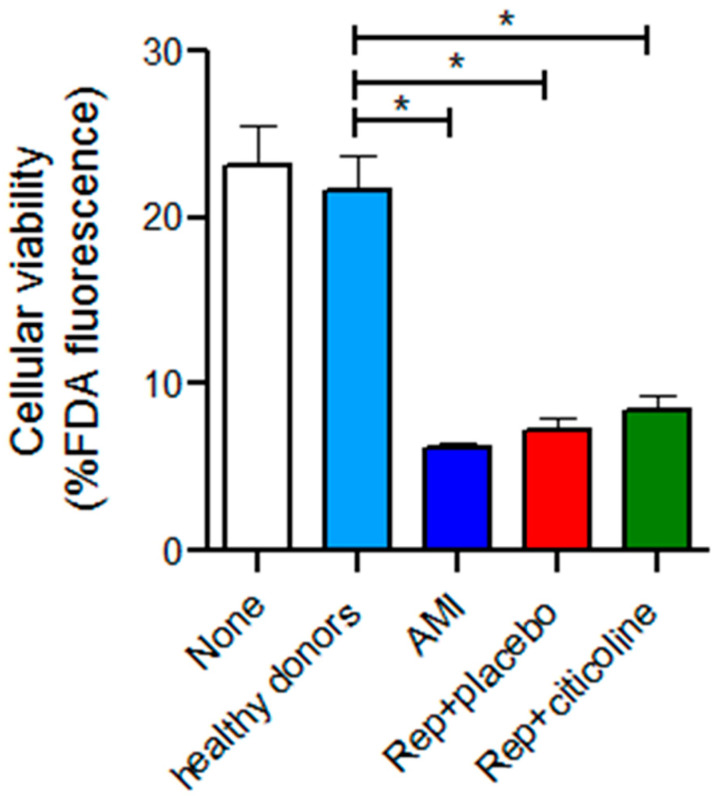
Effect of exosomes obtained from AMI patient plasma subjected to coronary angioplasty and treated with citicoline on cellular viability. Cellular viability was measured with fluorescein diacetate (FDA, *n* = 9 by duplicate). H9c2 cells exposed to hypoxia and reoxygenation were incubated for 24 h with exosomes from AMI patients and with exosomes from reperfused patients treated with and without citicoline. Results are shown as mean ± SE, * *p* ≤ 0.05.

To determine if the differential expression of miR-233-3p, miR21-5p, and miR-92 in exosomes might exert deleterious or cardioprotective effects on cells subjected to stress, we incubate H9c2 cells subjected to hypoxia and reoxygenation (H/R) with exosomes from IAM patients treated with and without citicoline. As observed in Figure 6, the hypoxic and reperfusion stress diminished cell viability to 23.1 ± 7.4%. While incubation with exosomes from healthy donors had no effect on viability (21.6 ± 6.3%), cell viability further diminished to 6.6 ± 1% in the presence of exosomes from infarcted patients and with exosomes from reperfused patients that received placebo (7.2 ± 2.1%). We did not observe improved viability in H9c2 cells incubated with exosomes from reperfused patients with citicoline treatment (8.49 ± 2.6%).

## 3. Discussion

This study was conducted to determine the prognostic value of circulating levels of exosomes in STEMI patients subjected to coronary angioplasty and to detect the possible changes that citicoline administration, before reperfusion, might exert on their protein and miRNA content. It is known that citicoline is a safe drug that has no adverse effects, even at 2000 mg per day, in human volunteers from different phase III clinical trials. For example, the US Citicoline Stroke Treatment Study IP302-018 was a 118-center, randomized, double-blind, efficacy trial in which citicoline was administrated 1000 mg PO twice a day to acute stroke patients. Due to the excellent safety profile of citicoline, it was suggested to continue the drug for an additional 6 weeks in such patients [20]. In the “ICTUS Study: International Citicoline Trial on Acute Stroke (ICTUS)”, published in 2012, citicoline was administrated for 3 days and then orally until complete after 6 weeks of treatment at a dose of 2000 mg per day. Patients with moderate to severe acute ischemic stroke treated with placebo or with citicoline showed similar adverse effects [21].

We found that EVs, particularly exosomes, augment early in STEMI patients before coronary angioplasty and decreased 24 h after reperfusion (Figure 2a). Conversely, troponin I and creatine kinase (CK-MB) levels were lower before reperfusion than after coronary angioplasty (Figure 1a,c). In this regard, several studies report that troponin I content is a useful marker in acute coronary syndrome diagnosis with a 95% global sensitivity, due to its nearly absolute myocardial tissue specificity and to its potential to reflect very small areas of necrosis [22]. According to Meraz-Soria et al. [23], troponin I levels increase in AMI cases with more than 4 h of evolution; therefore, the recommended interval to obtain samples that rule out or confirm myocardial infarction diagnosis is between 3 and 6 h as a first approximation, and again after 12–24 h if the first samples were negative and the clinical symptoms suggest AMI [24]. Our findings support the proposal that in a need of an early diagnosis, it would be desirable to detect exosome levels as a rapidly rising biomarker, followed by confirmation of a later response biomarker, such as troponin I.

Several strategies have been used to reduce the risk of further myocardial damage after elective primary angioplasty. In this sense, citicoline, a compound extensively used in patients with neurological disorders, including cerebral ischemia [25], has shown cardioprotective effects in isolated cells and murine models of cardiac reperfusion damage. Citicoline administration diminishes reperfusion-induced ventricular arrhythmias, decreases oxidative stress, and apoptotic cell death [15], besides preserving mitochondrial function [14]. In this work, we evaluate the association between citicoline and changes in exosome-derived proteins or miRNAs associated with cardioprotection. As virtually all body cells secrete extracellular vesicles, we expected to obtain a heterogeneous vesicle population. Monocytes, leucocytes, platelet, endothelial, and cardiomyocyte-derived exosomes were detected; although, we cannot rule out that other cells deliver exosomes to the circulation after the ischemic/reperfusion event.

We found that CD146+ increases in exosomes from patients treated with citicoline during the reperfusion procedure in comparison with exosomes from reperfused patients that received placebo and with healthy donors (Figure 3c). Similar results were reported by Jeanneteau et al., who observed a significant increase in endothelium-derived CD146+ microparticles in healthy donors that received a remote conditioning procedure employing inflation and deflation of a blood pressure cuff placed around the arm [26]. They also reported that remote conditioning increased endothelium-derived microparticles from healthy rats; although, they failed to exert cardioprotection in infarcted rat hearts. On the other hand, we show that microparticles carrying tissue factor (CD142) diminish significantly in reperfused patients treated with citicoline, as compared with the group that received a placebo (Figure 3d). CD142 is the initiator of the extrinsic coagulation cascade and plays a key role in intravascular thrombus formation. Some reports show that circulating microparticles are the main reservoir of CD142 activity in plasma [27] and that this circulating factor might predict atherothrombotic risk.

Extracellular/circulating miRNAs contained in microvesicles and exosomes serve as biomarkers for diseases and have relevant roles in intercellular communication. miRNAs regulate the activity of host cells but are also secreted and transferred to recipient cells [28]. We observed high levels of miR-223-3p in exosomes during AMI and after reperfusion (Figure 4a), which confirms previous studies of miRNA expression profiles in animal models subjected to ischemia/reperfusion [29]. We also found that such levels decrease in exosomes from reperfused patients treated with citicoline. Due that most targets of miR-233-3p are related to inflammation (i.e., NLRP3, IKK, and interleukin (IL)-1, IL-6, Roquin, and Pknox1 [30]), it is tempting to speculate that citicoline might diminish the inflammatory response by inhibiting the NLRP3 inflammasome or other components of this pathway. In this respect, we previously reported that citicoline diminished tumor necrosis factor-alpha (TNF-α) and increased the pro-resolving lipid mediator resolvin D1 in ischemic-reperfused rat livers [31].

The role of miR-21 in reperfused myocardium is well documented. It has been described as being up-regulated in mice heart subjected to the cardioprotective maneuver of ischemic preconditioning [32], and that transgenic mouse overexpressing cardiac miR-21 show resistance to I/R myocardial injury [33]. miR-21-5p targets different pro-apoptotic genes, activating prosurvival signaling pathways on cardiomyocytes [29]. In this regard, the silencing of miR-21-5p with antagomiR is accompanied by the hydrogen peroxide-triggering of necrosis and apoptosis in cardiomyocytes; however, our results show decreased levels in the citicoline-treated group.

On the other hand, the diminution of miRNA-92 content in exosomes from citicoline-treated patients as compared with patients who received placebo (Figure 4c), is consistent with studies in which the inhibition of miR-92 enhanced cardiac cell proliferation and reduced apoptosis after AMI [34]. Moreover, it matches with reports in which anti-miR-92a improved the recovery of cardiac function in both murine and pig models [35,36]. Even more, it was reported that citicoline reduces apoptosis induced by oxidative stress after hypoxia/reperfusion in cardiomyocytes [13]. Overall, the changes observed in miR-233-3p and miRNA-92 content in exosomes suggest the activation of pathways related to cardioprotection in association with citicoline administration. However, more studies are needed to establish a clear association between dysregulated miRNAs and myocardial post-infarct outcomes in the presence of citicoline.

We also sought to determine if this compound induces changes in the exosomes’ protein cargo, particularly in proteins with important roles in the selective transport of miRNAs into EVs. It has been reported that Cav-1 is relevant to miRNAs transfer into extracellular vesicles, besides it being a necessary component in the biogenesis of such particles during cell oxidative stress and that the process is facilitated by proteins such as hnRNPA2B1 [37]. Extracellular vesicles containing Cav-1 are related to tumor development and progression [38]; in addition, cav-1 levels increase in exosomes from melanoma serum as compared to exosomes from healthy individuals [39]. To our knowledge, there are no reports addressing whether EVs containing caveolins are critical to confer cardioprotection. However, a few years ago it was suggested that the loss of helium-mediated cardioprotection in isolated hearts, as compared with that observed in an in vivo model, in which both Cav-1 and Cav-3 were overexpressed, might be due to the lack of circulating factors [40,41]. On the other hand, Villarroya-Beltri et al. [42] showed that hnRNPA2B1 and hnRNPA1 binding to specific motifs within the sequence of miRNA determines their localization in either the cytoplasm or EVs. However, we did not find differences between Cav-1, Cav-3, or hnRNPA2B1 content in the evaluated groups (Figure 5), which suggests that other proteins or mechanisms might be associated with the citicoline-induced modified expression of miR233-3p, miR92, and miR21-5p in exosomes.

Limitations of this study are that we do not have a complete scenario of the outcomes of the patients and that no functional association was found between exosomes from citicoline-treated patients and cardioprotection, which might result from inadequate incubation time or that miRNA concentration in exosomes was too low to exert an effect. We are also aware that the Western-blot images are not as good as we expected; however, no more samples are further available, but the images provided are adequate to draw firm conclusions. On the other hand, in accordance with recent reports, we use miR-16-5p as a reference gene in exosomes [43,44] instead of stable small RNA controls, as there is evidence that, for serum miRNAs, the above-mentioned small RNAs might be highly variable or not stably detectable [45]. Finally, it would be interesting to evaluate the effect of exosomes from citicoline-treated patients on differentiated cardiac-like cells, and not only in H9c2 myoblasts; nevertheless, this represents a first approximation to pave the way for a more suitable cell model.

## 4. Materials and Methods

### 4.1. Antibodies and Reagents

Anti-CD81 (sc-166029), anti-CD9 (sc-13118), anti-Hsp70 (sc-32239), and anti-hnRNPA2/B1 (sc-53531) antibodies were from Santa Cruz Biotechnology (Dallas, TX, USA). From Abcam (Cambridge, MA, USA) were anti-caveolin-1 (Cav-1, ab2910) and anti-caveolin-3 (Cav-3, ab2912). The antibodies used in flow cytometry to characterize the origin of exosomes were anti-human CD142-PE to monocytes and endothelial cells (#550313), anti-human CD146-PE-CF594 to endothelial cells (#564327), anti-human CD45-APC (#555485) to leucocytes and hematopoietic cells, and anti-human CD36-PerCP-Cy (#561536) to platelets from BD Biosciences (San Jose, CA, USA). Hsp70 was used as a specific marker of cardiomyocyte-derived exosomes [46]. The Megamix-Plus FSC Beads were acquired from BioCytex (BioCytex, Marseille, France) and aldehyde/sulfate latex beads with 4 μm (A37304) were from Invitrogen™ (Waltham, MA, USA), other reagents used are mentioned where required.

### 4.2. Group of Patients

This study included 56 patients admitted to the intensive care unit of the Instituto Nacional de Cardiología (Mexico City, Mexico) suffering from ST-segment elevation myocardial infarct (STEMI) and who were candidates for primary angioplasty, between April 2015 and November 2018 (Approval number 14-886 of the Institutional Ethics Committee for protocol, “Cardioprotective effect of intravenous citicoline in patients subjected to primary angioplasty”, available in Supplementary Material Tables S1 and S2). These patients (aged 18–70 years) had no more than six hours of pain onset, occlusion of a major artery in the proximal segment, and TIMI = 0. Patients with ventricular fibrillation, active blooding, chronic renal insufficiency, and those with thrombolytic therapy were excluded from the study. Blood samples (4 mL) were obtained at their income and 24 h after primary angioplasty. Half of such patients randomly received 2 g per day of citicoline: 1 g was administrated during primary angioplasty and then, 1 g every 12 h by intravenous infusion for the first 5 days, whereas the other patients received placebo. The dose of citicoline was based on that reported in the International Citicoline Trial on acute Stroke (ICTUS) trial [21]. Citicoline was administrated in patients who, at their income, referred no more than six hours of pain onset, i.e., the ischemic episode. The procedure delay time was 3.23 ± 2.13 h in the citicoline group and 4.06 ± 2.02 h in the placebo group. Overall, the procedure delay time was 3.45 ± 2.08 h.

In addition, blood samples from healthy donors (without a background of AMI) were obtained from the blood bank of our institution and were used as a control group. All patients signed informed consent and their characteristics are listed in Table 1.

### 4.3. Obtention of Exosomes from Healthy Donors and Patients Subjected to Angioplasty

Blood was collected in centrifuge tubes containing ethylenediaminetetraacetic acid (EDTA) and processed immediately at room temperature to obtain plasma. Plasma samples from 26 patients (13 with citicoline treatment and 13 who received placebo) along with plasma samples from 26 healthy donors were used to obtain exosomes using the Total Exosome Isolation Kit from plasma (Invitrogen™, Waltham, MA, USA), according to the manufacturer’s instructions, then stored at −70 °C until use. Protein was determined by the Lowry method [47]. The approval to evaluate exosomal content in plasma samples from these patients was granted by the Institutional Ethics Committee, with number 20-1152 for protocol, “Evaluation of citicoline administration on exosomal caveolin content in patients with acute myocardial infarction subjected to angioplasty” in February 2020.

### 4.4. Characterization of Exosome-Enriched Fractions

Exosome-enriched fraction was characterized by immunodetection of tetraspanins CD9 and CD81 (see below 4.8 Immunoblotting), by measuring the activity of acetylcholine esterase (an enzyme marker of exosomes), by transmission electron microscopy, and by nanoparticle tracking analysis (NTA).

Acetylcholine esterase was evaluated according to Ellman et al. with brief modifications [48]. Briefly, 20 µL of exosomes was resuspended in 110 µL of phosphate-saline buffer (PBS, pH 7.4) and 37.5 µL of each diluted sample, and added to 96-well microplates. Acetylthiocholine (1.25 mM) and 5-dithiobis-2-nitrobenzoate (DTNB, 0.1 mM) were then added to the exosome fractions in a final volume of 300 µL. The rate of production of thiocholine produced by acetylthiocholine hydrolysis and its reaction with the thiol-reagent DTNB was measured at 412 nm every 5 min for 30 min. The activity is expressed as moles substrate hydrolyzed/min per mg protein, using an extinction coefficient of 1.36 × 104 Lmol^−1^/cm^−1^ for DTNB.

Transmission electron microscopy (TEM) was performed by pouring directly 30 µL of the exosome samples onto FCF-200-NI coated grids and counterstained with uranyl acetate and lead citrate. The grids were air-dried and samples were examined with an FEI Tecnai transmission electron microscope (Hillsboro, OR, USA). Particles were measured using the multi-measure ROI tool of ImageJ 1.48 software (NIH, Bethesda, MA, USA), from digital images obtained at similar magnifications.

NTA was evaluated in a Nanosight NS300 microscope (Malvern Panalytical, Malvern, UK) using a 405 nm laser beam. Samples were diluted at 1:50 in pre-filtered PBS and injected into the sample chamber. NTA predicts the size distribution of particles in a liquid suspension based on their light-scattering properties and Brownian motion. Triplicate videos of 30 s were recorded for each sample and Nanosight software was used to identify individual particles on a frame-by-frame analysis, rendering particle size distribution and concentration of the sample.

### 4.5. Flow Cytometry

Fluorescence-activated cell sorting (FACS) analysis was used as described by Cai et al. [49] with slight modifications. Briefly, a final concentration of 5 µg/mL of exosomes was incubated overnight with 5 µL 4 µm diameter aldehyde/sulfate latex beads with gentle shaking in PBS (pH 7.4) in a 10 μL final volume. The mixture was blocked with 4% bovine serum albumin for 1 h, and then the exosome-coated beads were centrifuged at 17,000 × g for 30 s, washed with PBS under the same conditions, and resuspended in 50 µL of PBS. Afterward, beads were incubated in the dark with an antibody cocktail (antibodies to human CD142-PE, CD146-PE-CF594, CD45-APC, and CD36-PerCP-Cy) or with PBS in a 50 µL final volume for 1 h. Each antibody was titrated and the dilution with the brightest staining and minimum background was chosen. Subsequently, labeled beads were washed with PBS and centrifuged at 17,000× *g* for 30 s. The supernatant was discarded and labeled beads were resuspended in 400 µL PBS and analyzed using a BD FACSAria Fusion Flow Cytometer (Becton Dickinson, Mountain View, CA, USA) and FlowJov10.8.1 software. Results are reported as positive events (%) for each antibody used.

### 4.6. Isolation of miRNAs from Plasma Exosomes

RNeasy serum/plasma midi kit (Qiagen, Hilden, Germany) was used for exosome RNA isolation. During RNA purification, cel-miR-39 spike-in control (Qiagen, Hilden, Germany) was added according to the manufacturer’s recommendation. The RNA isolated from the exosomes was transformed to cDNA, as described below.

### 4.7. miRNAs Determination by RT-qPCR

miRNAs were determined as previously described [50] with minor modifications, using two-step RT-qPCR with RT-primer specific assay in combination with TaqMan probes (Applied Biosystems™, CA, USA): miR-39 (Assay ID: 000200), miR-21-5p (Assay ID: 000397), miR-155-5p (Assay ID: 002623), miR-223-3p (Assay ID: 002295), and miR-92-5p (Assay ID: 000430). Firstly, 2 µL from eluted RNA was reverse-transcribed using the TaqMan MicroRNA Reverse Transcription Kit (Applied Biosystems™, CA, USA). RT-reaction consisted of 30 min at 16 °C, 30 min at 42 °C, and 5 min at 85 °C in a Veriti Thermal Cycler (Applied Biosystems™, CA, USA). Then, samples of cDNA (1.5 µL) were amplified in a final volume of 10 µL. PCR reaction was performed in a CFX96 System qPCR Instrument (Bio-Rad, CA, USA) included an initial denaturation step at 95 °C for 10 min, followed by 45 cycles of 95 °C for 15 s, 60 °C for 60 s, and 72 °C for 1 s. miRNAs relative concentrations were normalized with Ct values of cel-miR-39, and values were calculated using the 2−ΔCt formula. All Ct values for cel-miR-39 ranged from 20 to 22 cycles both for total plasma and for exosomes’ RNA isolation. Evaluated miRNAs were miR-233, miR-21, miR-92, and miR-155, on the basis of an extensive bibliographic search in the public database PubMed (https://pubmed.ncbi.nlm.nih.gov/, accessed on 1 April 2022) [51] regarding their association with ischemic myocardium and reperfusion injury.

### 4.8. Western-Blot Analysis

Plasma-derived exosomes were lysed using ice-cold RIPA buffer (50 mM Tris-HCl, pH 7.6, 150 mM NaCl, 1% NP-40, 0.5% sodium deoxycholate, 0.1% SDS) supplemented with 1 mM PMSF and protease inhibitor (SigmaFast, Sigma-Aldrich, St. Louis, MO, USA). Proteins (20 μg) of each sample were diluted 1:1 with Laemmli buffer supplement with dithiothreitol (50 mM) and denatured by boiling for 5 min. Proteins were separated on 12% SDS-PAGE gels and transferred to a PVDF membrane (Immobilon^®^-P, Millipore, Billerica, MA, USA). Membranes were incubated with specific primary antibodies: anti-CD81 (1:1000), anti-CD9 (1:1000), anti-Hsp70 (1:1000), anti-caveolin-1 (1:1000), anti-caveolin-3 (1:1000), and anti-hnRNPA2B1 (1:750) with milk at 3% overnight at 4 °C. Posteriorly, HRP-conjugated secondary antibody was used (1:15,000, Jackson ImmunoResearch Lab., West Grove, PA, USA) for 1 h at room temperature with constant agitation. The immunoblotted proteins were detected with Immobilon™ Western Chemiluminescent HRP Substrate detection system (Millipore, Billerica, MA, USA). All images were analyzed using ImageJ (NIH, Bethesda, MA, USA) and reported as arbitrary units.

### 4.9. Cell Culture and Model of Hypoxia/Reoxygenation (H/R)

Rat cardiomyoblasts cell line (H9c2, CRL-1446) was purchased from ATCC^®^ (Manassas, VA, USA) and maintained in a complete medium containing Dulbecco’s modified Eagle’s medium (DMEM, Gibco, Grand Island, NY) in high glucose supplemented with 10% fetal bovine serum (Biowest, Riverside, MO) plus antimycotic–antibiotic solution (Gibco, Grand Island, NY), incubated at 37 °C in a humidified incubator with 5% CO_2_. Fifty microliters of exosomes (20 μg/mL) were incubated with 2 × 10^3^ cells/well in a 96-well-plate for 24 h before the induction of H/R. After, the cells in serum-free and glucose-free DMEM were placed in a hypoxic chamber (Billups-Rothenberg Inc., CA) containing 5% CO2, 0.1% O_2_, and 94% N_2_ for 24 h. Subsequently, cells were exposed to reoxygenation in standard conditions (95% air/5% CO_2_) in a complete medium for 1.5 h.

### 4.10. Cellular Viability

Cells (2 × 10^3^/well) were incubated with 12 μM fluorescein diacetate for 5 min at 37 °C in the dark and fluorescence intensity was quantified on a Synergy multi-modal plate reader (BioTek, Winooski, VT, USA) using the following filters: excitation 485/20 nm and emission 528/20 nm. Cell viability was reported as a percentage and normalized to the control group.

### 4.11. Statistical Analysis

Results are reported as mean ± standard error of the mean (SEM). Differences in a single variable, such as caveolin-1, caveolin-3, and hnRPA2/B1 immunodetection in plasma exosomes are compared among groups by one-way ANOVA. Other results were analyzed with a Kruskal–Wallis non-parametric test followed by Dunn’s multiple comparisons analysis. Data from flow cytometry are expressed as mean ± SD and analyzed with the Mann–Whitney U or Wilcoxon test. For all tests, we used Prism 6.0 software (GraphPad, San Diego, CA, USA), and results with a *p*-value ≤ 0.05 were considered statistically significant. Raw data and uncropped western blots are available in supplementary material Tables S1 and S2.

## 5. Conclusions

We show here that exosomes from infarcted patients act as damage markers and exacerbate injury in cells subjected to hypoxia and reoxygenation. Although we did not find that exosomes from citicoline-treated patients trigger a repair response, this compound induces changes in specific miRNAs related to cardioprotection.

## Figures and Tables

**Table 1:** Patient characteristics and risk factors.

	Rep + Placebo	Rep + Citicoline
Age (year)	56.38 ± 10.57	53.63 ± 8.11
Corporal mass index	27.75 ± 4.709	27.85 ± 3.594
Left ascending artery	20%	25%
Right coronary artery	31%	24%
Hypertension	10.3%	25.9%
Obesity	58%	77%
Diabetes	14%	20.7%
Smokers	27.5%	37%

## Data Availability

Data is contained within the article and supplementary material.

## Supplementary Materials

The following are available online at https://www.mdpi.com/article/10.3390/ph15080925/s1, Table S1: RAW DATA figure 4, Table S2: RAW DATA.
